# Pneumobilia Caused by Blunt Abdominal Trauma

**DOI:** 10.5334/jbsr.1661

**Published:** 2019-01-02

**Authors:** Hadrien Fourneau, Charlotte Grandjean

**Affiliations:** 1 Cliniques Universitaires St Luc, BE; 2 Cliniques St Pierre Ottignies, BE

**Keywords:** Pneumobilia, abdominal trauma, sphincter of Oddi, duodenum diverticulum

A 70-year-old man was admitted to the emergency department after a car accident. He was driving at 50 kilometers per hour and was not wearing a seatbelt. There was no clinical sign of thoracic or abdominal lesion.

Initially, only a cerebral computed tomography (CT) was performed and did not show any lesion, but the clinical course was marked by sepsis, hypotension and confusion.

Later on, a contrast-enhanced thoraco-abdominal CT was carried out (Figure 1) and showed liver laceration (asterisks) that involved the middle hepatic vein, subcapsular liver hematoma (white crosses), haemoperitoneum, rib fractures and pneumobilia (arrows). There was also a large duodenal diverticulum (DD).

**Figure 1:**
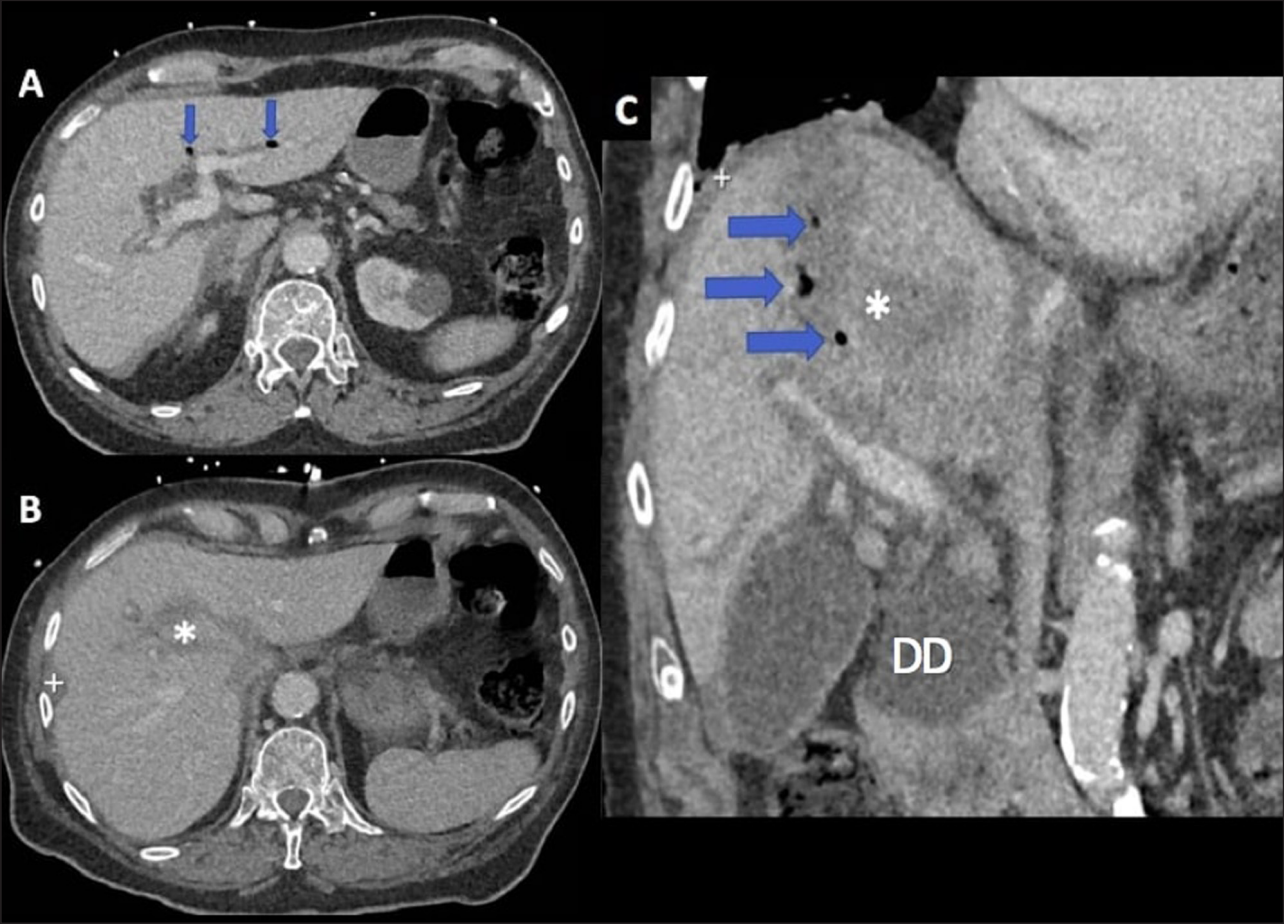


The patient was conservatively managed in intensive care unit. The clinical and radiological outcome was good, with progressive regression of hepatic traumatic lesions and pneumobilia until the last follow-up CT at day 75 (Figure 2).

**Figure 2:**
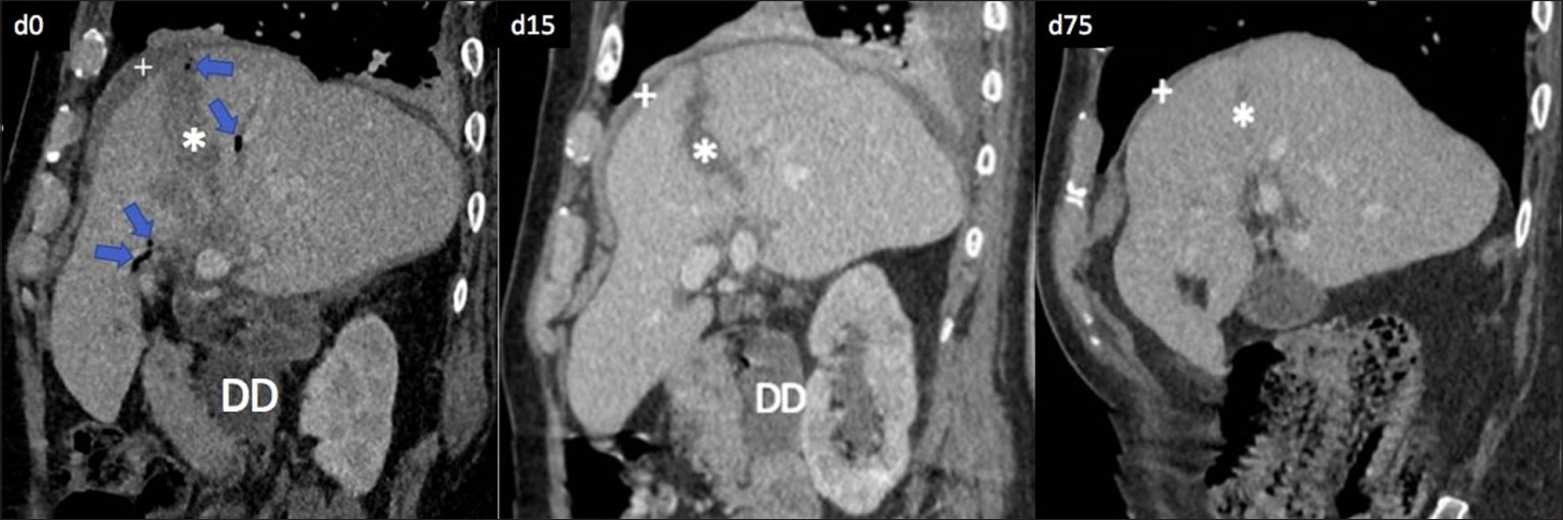


## Comment

Pneumobilia is the presence of gas in the biliary tree. It may be detected with plain X-ray and ultrasound (hyperechoic signal in the biliary system with acoustic shadowing artefact), but it is better characterized by CT.

It is a common finding after interventions on the bile duct (endoscopic biliary manipulations, biliary-enteric anastomosis or percutaneous transhepatic cholangiography). Other common causes of pneumobilia included emphysematous cholecystitis, biliary tract infection, liver abscess, biliary-enteric fistula (gallstone erosion or peptic ulcer for example) and high gastrointestinal obstruction.

Less frequently, pneumobila can occur in the context of an abdominal blunt trauma. The sphincter of Oddi can support a pressure as high as 60 cmH_2_O, which usually prevents retrograde air flow in the common bile duct. Rare cases of pneumobilia secondary to abdominal trauma causing high elevation of abdominal pressure (>60 cmH_2_O) have been reported. There have been fewer than 10 cases to our knowledge [1]. We believe that this condition can explain the air in the biliary tract of our patient. Nevertheless, the sphincter of Oddi may be incompetent, secondary to drugs effect (atropine for example) or sphincter lesions, but it can also be constitutional. The duodenum diverticulum may have contributed to pneumobilia because of the gas trapped inside and its potential (though controversial) implication in the dysfunction of the sphincter of Oddi.
